# Efficacy of an educational website on headaches in schoolchildren: A cluster‐randomized controlled trial

**DOI:** 10.1111/head.14923

**Published:** 2025-03-14

**Authors:** Henrike Goldstein, Lisa‐Marie Rau, Clarissa Humberg, Verena Bachhausen, Lorin Stahlschmidt, Julia Wager

**Affiliations:** ^1^ Department of Children's Pain Therapy and Paediatric Palliative Care, Faculty of Health, School of Medicine Witten/Herdecke University Witten Germany; ^2^ German Paediatric Pain Centre Children's and Adolescents' Hospital Datteln Datteln Germany; ^3^ PedScience Research Institute Datteln Germany

**Keywords:** headache, intervention, longitudinal, pediatric, randomized controlled trial, school

## Abstract

**Objective:**

In this cluster‐randomized controlled trial, we developed an educational website on tension‐type headache and migraine for children and adolescents and evaluated its effectiveness in a school setting.

**Background:**

Primary headaches are a widespread issue in children and adolescents, often persisting into adulthood and associated with considerable disabilities, costs, and reduced quality of life. Effective management of primary headaches may prevent chronicity and its associated consequences.

**Design:**

Guided by a workbook, *N* = 814 fifth and sixth grade students explored the website during class. Data were collected before the headache education and at three further assessments, each 4 weeks apart, between November 2021 and April 2022. Participants were randomly assigned by class to either the intervention group, which received the website‐based educational intervention after the first data collection, or a control group, which accessed the website after the last data collection.

**Results:**

The intervention significantly increased children's headache‐related knowledge (time × group interaction: *β* = 0.35, 95% confidence interval [CI] = [0.30; 0.41], *p* ≤ 0.001) and resulted in fewer passive pain coping strategies (time × group interaction: *β* = −0.06, 95% CI = [−0.12; 0.00], *p* = 0.044). However, the intervention did not significantly reduce the number of days with headaches, use of headache medication, or school absences due to headaches.

**Conclusion:**

While the website is an effective educational tool for imparting knowledge about headaches, even initiating small behavioral changes, it does not lead to substantial changes in behavior or headache characteristics. Educating children via this website may lay a solid foundation of knowledge, but the intervention should be expanded and supplemented with closer supervision to achieve more significant behavior changes and improved outcomes.

## Plain Language Summary

Many children have headaches, so we wanted to find out if a headache‐focused educational website and workbook could improve headaches and their management in school children. Participants were 814 fifth and sixth graders who were randomly assigned to the intervention group or the control group. We found that the intervention increased knowledge and reduced passive coping strategies, but did not change headache symptoms or behaviors; therefore, this intervention may be an easily accessible, cheap, and time‐saving option for schools to impart knowledge on headaches for their students.

**Abbreviations**
CFI: comparative fit index
CG: control group
CI: confidence interval
IG: intervention group
PedMIDAS: pediatric migraine disability assessment
PPCI‐r: Pediatric Pain Coping Inventory–Revised
RCT: randomized controlled trial
RMSEA: root mean square error of approximation
SD: standard deviation
SRMR: standardized root mean square residual

## INTRODUCTION

Numerous epidemiological studies in recent decades have shown that headaches, specifically primary headaches, are widespread and increasing among children and adolescents.^1^, ^2^ These headaches often persist into adulthood, causing long‐term health‐care costs.^3^, ^4^ Pain education is a crucial first step in preventing headache chronification, as it promotes health‐positive behaviors.^5^ A systematic review in adults showed that migraine education can reduce headache frequency, lessen headache‐associated functional impairment, and improve quality of life.^6^ For children, schools are well suited for implementing headache prevention programs, given their broad reach.^7^ Studies in this setting show that educational videos on headache or pain can boost children's knowledge, especially among those affected by pain, though these videos alone do not affect pain characteristics or behaviors.^8^, ^9^ Booster sessions have shown to be more effective than one‐time interventions of pain neuroscience education.^10^ Based on these findings, it appears promising to create health‐promoting resources that can be easily delivered in class. We, therefore, developed a website on primary headaches for children and adolescents, aiming to impart knowledge on tension‐type headache and migraine, headache management strategies, and prevention.

This study aimed to evaluate the effectiveness of a guided, website‐based headache education intervention on schoolchildren's behavior (including headache‐related school absences, medication use, and coping strategies), as well as headache characteristics, knowledge, and pain self‐efficacy using a cluster‐randomized controlled trial (RCT). We hypothesized that children in the intervention group (IG) would demonstrate improved outcomes 3 months after the initial introduction of the website compared to the control group (CG), who did not have access to the website. We further hypothesized that children with recurrent headaches or who revisited the website would benefit more than those without headaches or who did not revisit. Finally, we investigated whether increased knowledge could influence health behavior, as suggested by the Integrated Theory of Behavior Change.^11^ Kisling et al. found promising evidence in a study with comparable design and sample, prompting us to replicate their model on how behavior mediates the association between headache knowledge and intensity.^8^

## METHODS

### Design

Data were collected at six schools in North Rhine‐Westphalia, Germany, as part of the CHAP study (“Chronic Headache in Adolescents: The Patient Perspective on Health Care Utilization,” Reference Number: 01GY1615). This is the primary analysis of these data. The study was preregistered in the German Clinical Trials Register (ID: DRKS00027065) and included different types of German secondary schools (Realschule, Gesamtschule, and Gymnasium). This RCT involved four assessments conducted 4 to 5 weeks apart between November 2021 and April 2022. The first and the last assessments took place in the classroom (T1 and T4), while the second and third were conducted online (T2 and T3). Participants received 2 Euros per survey or 10 Euros for completing all four surveys, given as vouchers or charitable donations.

### Procedure

All fifth‐ and sixth‐grade students from the six schools were invited to participate in the study. To ensure that more severely impaired children had the opportunity to participate, study information and consent forms were distributed to absent students, with several catch‐up appointments for those who missed data collection.

The 44 school classes (each representing one cluster) were randomly assigned to either the IG or CG in a 1:1 ratio, stratified by school type, resulting in 22 classes per group (masking was not feasible). Students in the IG took part in the intervention about 2 weeks after T1, whereas the CG received access to the intervention after data collection was complete (waitlist control design). The intervention was integrated into the school curriculum delivered preventively even to students not participating or not yet affected by headaches, considering the increasing headache prevalence with age.^12^ Data were collected only from those who assented and whose parents provided informed written consent.

### Guided website‐based headache education

The website‐based intervention comprised the newly developed educational website on headaches, meine‐kopfsache.com (English website: headeggs.org), and an accompanying workbook designed to help students explore the website (German version: https://www.meine‐kopfsache.com/wp‐content/uploads/rallye.pdf). The website underwent extensive pre‐testing with tertiary care patients and students, after which materials were thoroughly and substantially revised. The interactive website was created by headache experts from the German Paediatric Pain Centre in collaboration with patients on behalf of the self‐help group UVSD SchmerzLOS e.V., with financial support from the Techniker Krankenkasse health insurance fund. These institutions had no influence on the website content. The website provides evidence‐based, guideline‐compliant information on how to differentiate between tension‐type headache and migraine, along with strategies for managing and preventing headaches. It also includes general health tips on physical activity, sleep hygiene, stress relief, and relaxation, making it useful for all children. The website content is in accordance with the Internet Intervention Model,^13^ outlining features necessary for promoting behavioral change and symptom improvement (Supplement S1 in supporting information).

The website was hidden from search engines throughout the study period.

Students in the IG explored the website during a 90‐min lesson under the supervision of a project team member. A workbook guided them through each webpage to ensure thorough engagement with the content. After the initial session, children in the IG could revisit the website independently.

### Sample

**FIGURE 1:**
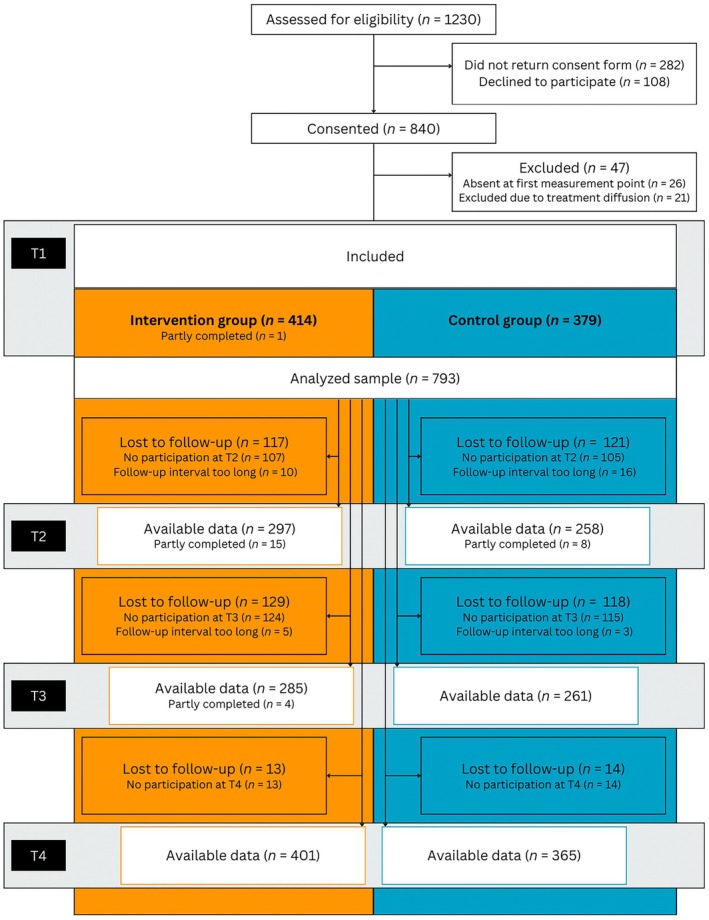
Flowchart. T1, T2, T3 and T4; First, second, third, and fourth assessment. [Colour figure can be viewed at wileyonlinelibrary.com]

Of 1230 approached students, 840 agreed to participate in the study (68.3%; Figure 1). Eligibility criteria for the study included: (1) sufficient German language proficiency, (2) informed written assent/consent from both students and parents, and (3) attendance at the first measurement point. Children were excluded if consent for study participation was withdrawn. At the first data collection, 814 students were present, of which 414 were in the IG and 400 in the CG. Baseline differences between the IG and CG were assessed using Welch's *t* test for continuous variables and chi‐squared tests (*χ* ^2^) for categorical variables (Table 1). Randomization was successful, as participants in the IG did not differ from those in the control group regarding demographic, headache‐related, or psychological characteristics (all Benjamini–Hochberg corrected *p*‐values > 0.05).^14^

**TABLE 1:** Baseline characteristics of IG and CG.

	Total	CG	IG	*p*	*p* adjusted
	*N* = 793	*n* = 379	*n* = 414		
Age, years; mean (SD)	10.7 (0.7)	10.7 (0.7)	10.7 (0.7)	0.510	>0.999
Sex (girl)*; *n* (%)	441 (55.6%)	213 (56.2%)	228 (55.1%)	0.804	0.950
Headache status*; *n* (%)				0.017	0.221
No headaches	413 (52.1%)	201 (53.0%)	212 (51.2%)		
Non‐recurrent headaches	252 (31.8%)	131 (34.6%)	121 (29.2%)		
Recurrent headaches	128 (16.1%)	47 (12.4%)	81 (19.6%)		

	Children with recurrent headaches	CG	IG	*p*	*p* adjusted
	*n* = 127	*n* = 47	*n* = 80		
Headache‐related school absence^a^, days; mean (SD)	1.7 (3.3)	1.5 (3.6)	1.8 (3.0)	0.642	0.927
Headache medication consumption^a^, days; mean (SD)	2.7 (5.3)	3.4 (7.0)	2.3 (3.9)	0.318	>0.999
Headache days^a^; mean (SD)	9.5 (7.6)	9.6 (7.8)	9.4 (7.5)	0.889	0.963
Maximum headache intensity (0–10) ^a^; mean (SD)	7.2 (2.1)	7.1 (2.0)	7.2 (2.1)	0.771	>0.999
Average headache intensity (0–10) ^a^; mean (SD)	5.9 (2.0)	5.8 (2.0)	6.0 (2.0)	0.628	>0.999

	All participants	CG	IG	*p*	*p* adjusted
	*N* = 793	*n* = 379	*n* = 414		
Headache knowledge (0–12); mean (SD)	6.3 (1.9)	6.4 (1.9)	6.3 (2.0)	0.280	>0.999
Pain self‐efficacy (0–44); mean (SD)	25.8 (8.9)	25.8 (9.1)	25.8 (8.8)	0.982	0.982
Passive pain coping (0–20); mean (SD)	12.0 (3.4)	11.9 (3.4)	12.0 (3.4)	0.623	>0.999
Positive self‐instructions (0–14), mean (SD)	6.8 (3.1)	6.6 (3.1)	7.0 (3.1)	0.134	0.871
Seeking social support (0–16), mean (SD)	4.2 (3.2)	4.1 (3.1)	4.3 (3.2)	0.461	>0.999

*Note*: Cells contain means (standard deviations) for numeric, absolute (relative) frequencies for categorical variables (indicated with an *). *χ* ^2^‐ and *t*‐tests were applied as appropriate (*p* were adjusted for multiple testing using Benjamini–Hochberg correction; R package *compareGroups*). Outcomes marked with ^a^were analyzed only for children reporting recurrent headaches. Recurrent headaches were defined as having headaches for longer than 3 months that occurred at least once a week within the last month. Headaches not meeting these criteria were classified as non‐recurrent. For children without headaches, logical imputation was performed by setting scores to 0 for the following outcomes: headache days, average and maximum headache intensity, days with headache‐related medication consumption, and school days missed. Possible scale ranges are provided in parentheses for each variable.

Abbreviations: CG, control group; IG, intervention group; SD, standard deviation.

To avoid treatment diffusion, data from 21 children in the CG who reported visiting the website were excluded from analysis. See Supplement S2 in supporting information for analyses including these children. In addition, measurement points were excluded if the interval between the completed online questionnaires was too long (i.e., >53 days). The final analyzed dataset included 793 children (55.6% girls; *M*_age_ = 10.7 years, *SD*_age_ = 0.7 years, range_age_ = 9–14 years; 49.4% fifth graders). At T2, data were available for 70.0% of the children, 68.9% at T3, and 96.6% at T4.

At baseline, 630 children reported experiencing pain in the past 4 weeks. The most frequently mentioned pain was headache (*n* = 380; 47.9%), with 128 children (16.1%) meeting the criteria for recurrent headaches (onset more than 3 months ago and occurring at least once a week within the last month).^15^, ^16^ See Table 1 for a detailed sample description at T1.

### Measures

Students self‐reported demographic characteristics (age, sex, grade) at baseline, indicating if and where they had experienced pain in the past 4 weeks. Those who reported experiencing pain answered additional pain‐related questions. Behavior including school absence, medication use and coping strategies, headache characteristics, and self‐efficacy were assessed at all measurement points. Headache‐specific knowledge was not evaluated at T3 to minimize testing effects^17^, ^18^ and participant burden. Students assigned to the IG also provided feedback on the website.

The anticipated primary outcome was headache‐related impairment, measured using the Pediatric Migraine Disability Assessment (PedMIDAS), adapted to a 30‐day assessment period.^19^, ^20^ However, due to a data collection error, not all questions were displayed, making it impossible to calculate the full PedMIDAS score. As a result, the primary outcome is the PedMIDAS item on headache‐related school absence, which was collected correctly across all assessments.

#### Questionnaires completed by students with headaches in the past 4 weeks

Children with headaches reported the number of days they were unable to attend school and the number of days they had to leave school early due to a headache within the past 4 weeks (maximum 20 school days). They were instructed to categorize each day as either a total absence or an early departure, but not both. Responses were summed to calculate the total number of missed school days.

Children with headaches also indicated their headache‐related medication consumption by reporting the number of days they took medication for headaches in the last 4 weeks (maximum 30 days). They also reported whether they had pain in the past 4 weeks and indicated the location of their pain. For headaches, they answered questions on frequency and intensity. Headache frequency was measured by the number of days the child experienced a headache during the last 4 weeks (maximum 30 days). Maximum and average headache intensity in the past 4 weeks were rated using numeric rating scales ranging from 0 = no pain to 10 = strongest pain.

#### Questionnaires completed by all students

Headache knowledge was measured using a study‐specific, rigorously pre‐tested 15‐item questionnaire, each with four response options and one correct answer. Item response theory analyses were conducted to ensure validity (data from *N =* 793 students at T1; three parameter logistic model; R package mirt).^21^ Three items were removed: two due to extreme discrimination parameters (*a* > 6, indicating item dependence),^22^ and one due to a high guessing rate (*g* = 0.60). The person parameters of the remaining 12‐item solution followed a normal distribution with acceptable item difficulties and discrimination (<|4|), low guessing rates (*g* < 0.08), and covered a broad range of abilities (range of item difficulty: *b* = −3.49 to 3.40). The questionnaire can be found in Supplement S3 in supporting information. Scores ranged from 0 to 12, with 1 point per correct answer.

Children also reported their confidence in managing pain using the Scale for Pain Self‐Efficacy.^23^ This tool consists of 11 items (e.g., I've got my pain under control), with responses recorded on a five‐point scale (0 = not true to 4 = true) and added up forming a sum score; higher values indicate greater self‐efficacy. Children who had not experienced pain in the past 4 weeks were instructed to imagine how they would cope if they did have pain. The internal consistency of the scale was satisfying (T1:*α* = 0.84; T2:*α* = 0.86; T3:*α* = 0.88; T4:*α* = 0.87).

All children reported their pain coping strategies using the Pediatric Pain Coping Inventory–Revised (PPCI‐r).^24^ It consists of three subscales: passive pain coping (10 items, e.g., When I am in pain or something hurts, I … go to bed; internal consistency: T1:*α* = 0.65; T2:*α* = 0.69; T3:*α* = 0.72; T4:*α* = 0.72), positive self‐instructions (7 items, e.g., …tell myself to be brave; T1:*α* = 0.71; T2:*α* = 0.74; T3:*α* = 0.79; T4:*α* = 0.79), and seeking social support (8 items, e.g., … squeeze someone's hand or something else; T1:*α* = 0.73; T2:*α* = 0.79; T3:*α* = 0.82; T4:*α* = 0.82). Response options were 0 = almost never, 1 = sometimes, and 2 = often. Scores were summed for each subscale. Children who reported not having pain in the past 4 weeks were instructed to use the PPCI‐r to indicate how they generally cope with pain when it occurs.

#### Feedback on the website

Students in the IG were asked whether they had revisited the website after the guided session. If so, they provided feedback about the website. This included rating their overall satisfaction at T2 (1 = very good to 6 = insufficient) and assessing the comprehensibility of the content in subsequent assessments (0 = I didn't understand anything to 4 = I understood everything). They also reported which recommendations they had implemented in an open response format. Students in the CG were asked at T4 if they had ever visited the website.

### Power calculation

The sample size for the study was calculated for the primary outcome to detect a moderate effect (*d* = 0.5)^25^ with 90% power in a subsample of children with recurrent headaches (~20%). With an expected response rate of 80% ^26^ and considering the clustered data (intraclass correlation coefficient of 0.03),^27^, ^28^ we required a total sample of 110 students with recurrent headaches.

### Data analyses

To analyze the intervention effect, preplanned multilevel model analyses were conducted for continuous variables to accommodate the hierarchical data structure (observations nested within students; “classroom” level modeling requirements^29^ on an additional level were not met). These models implemented an autocorrelative covariance structure and restricted maximum likelihood estimation (R package nlme).^30^ The sample size was considered large enough to produce a type‐I error close to 5%.^31^ Uncertainty intervals (equal tailed) and *p*‐values (two tailed) were computed using a Wald *t*‐distribution approximation. Intraclass correlation coefficients ranged from 0.310 and 0.615, indicating sufficient between‐person variance to justify multilevel modeling. Time was included as a continuous variable in multilevel models and as categorical for post hoc analyses, comparing follow‐up assessments (T2 and T4) to the reference category T1 (intervention effect). For group comparisons, the CG was set as reference category. Data for headache‐related knowledge, pain self‐efficacy, and pain coping included all children, while only children with recurrent headaches at T1 were included in the analyses of headache‐related outcomes (*n* = 128).

Following the initial data examination, we analyzed whether the intervention was more effective in children with recurrent headaches regarding headache‐related knowledge, pain self‐efficacy, and pain coping. Children with non‐recurrent headaches at baseline were excluded from this analysis. Those with recurrent headaches at baseline were compared to those without headaches as a reference category in a three‐way interaction of time × group × headache status.

To investigate whether renewed engagement with the website led to improved outcomes, the initial models contrasted children in the IG who reported revisiting the website in the past 4 weeks at T3 or T4 with those in the IG who had not, using the latter as the reference category.

To gain insight into the relationship between headache‐related knowledge at T2 and average headache intensity at T4, we conducted a mediation analysis with children reporting headaches at T1 (*n* = 380). We hypothesized that this relationship could be mediated by headache‐related behavior at T3, using the manifest variables passive pain coping, days with medication use, and missed school days, following the approach of Kisling et al.^8^ Model fit was assessed using several fit indices, including comparative fit index (CFI), root mean square error of approximation (RMSEA), and standardized root mean square residual (SRMR).

The statistical significance level was set to *p* = 0.050. Hypothesis testing was two tailed. For exploratory group comparisons and post hoc tests, *p*‐values were adjusted using Benjamini–Hochberg correction.^14^ Effect sizes were interpreted according to Cohen.^25^ Data were processed using SPSS version 29 (IBM Corp., Armonk, NY, USA)^32^ and analyses were performed using R version 4.2.1 (R Foundation for Statistical Computing, Vienna, Austria)^33^ within the RStudio 2022.12.0 environment (Posit PBC, Boston, MA, USA,^34^ R packages detailed in Supplement S4 in supporting information).

### Ethics

The study was approved by the ethics committee of Witten/Herdecke University (reference number 268/2020).

## RESULTS

### Students' evaluation of the intervention

The website‐based intervention was successfully implemented across the 22 IG classes. At T2, students rated their overall satisfaction with the website at an average of 1.72, indicating a score between 1 (very good) and 2 (good) (SD_rating_ = 0.73; *n* = 282). The average comprehensibility score was 3.17 (standard deviation [SD] = 0.70), lying between 3 (I understood a lot) and 4 (I understood everything). At T3, 38.6% (*n* = 110) of the IG participating in this assessment reported revisiting the website in the past 4 weeks. Those who revisited the website rated its comprehensibility at 3.25 (SD = 0.65). At the last measurement point, 40.4% (*n* = 162) reported revisiting the website in the last 4 weeks, rating comprehensibility as 3.18 (SD = 0.77). Of those, 58.6% (*n* = 95) reported that they had implemented recommendations from the website. The most commonly cited tips included using the “bounce test” to distinguish headache types (*n* = 33), resting during migraine (*n* = 8), and increasing exercise (*n* = 7).

### Effectiveness of the website‐based education

**FIGURE 2:**
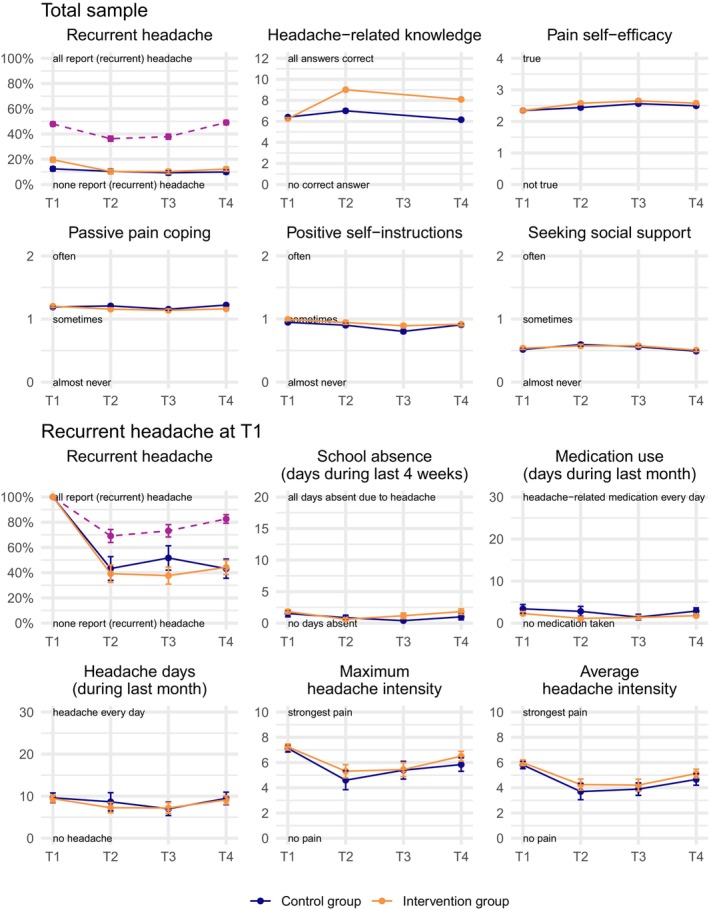
Mean trajectories of outcomes stratified by group (intervention vs. control group). Assessments occurred before the intervention (T1), and 4, 8, and 12 weeks after T1 (T2–T4). Error bars represent 95% confidence intervals. The upper panel displays data from the total sample (*N =* 793), while the lower panel shows data from children reporting recurrent headache at T1 (*n* = 128). Recurrent Headache: Headaches were classified as recurrent if they occurred on a weekly basis during the past month and persisted for more than 3 months in total.^15^, ^16^ Purple dashed lines indicate the total prevalence of headache (recurrent and non‐recurrent) in the displayed sample. Headache‐Related Knowledge: Study‐specific headache knowledge questionnaire consisting of 12 items. Each correct answer earns one point, contributing to a sum score (range: 0–12), with higher scores reflecting greater knowledge about headaches. Pain Self‐Efficacy: Scale for Pain Self‐Efficacy (SPaSE),^23^ with higher values indicating greater self‐efficacy. This instrument comprises 11 items (e.g., I've got my pain under control), each rated on a five‐point scale (0 = not true to 4 = true). Passive Pain Coping, Positive Self‐Instructions and Seeking Social Support: Subscales of the Pediatric Pain Coping Inventory–Revised (PPCI‐r)^24^ with higher values expressing a stronger manifestation. They consist of 10, 7, and 8 items, respectively (e.g., When I am in pain or something hurts, I … go to bed). Each item is rated on a three‐point scale (0 = almost never, 1 = sometimes, 2 = often). For Pain Self‐Efficacy, Passive Pain Coping, Positive Self‐Instructions, and Seeking Social Support, mean responses are displayed to ease interpretation. School Absence: Number of days in the past 4 weeks when attending school was either impossible or had to be cut short (max. 20 days). Medication Use/Headache Days: Number of days in the past 4 weeks with headache medication use/headaches (max. 30 days). [Colour figure can be viewed at wileyonlinelibrary.com]

The following outcomes were analyzed for participants with recurrent headaches in the past 4 weeks. No statistically significant main or interaction effects emerged regarding school absence due to headaches or days with headache‐related medication intake (Table 2, Figure 2). Additionally, there were no statistically significant effects found for the number of days with headaches, maximum headache intensity, or average headache intensity among children with recurrent headaches at baseline. The intervention did not influence the prevalence of recurrent headaches, as there were no significant main or interaction effects observed.

**TABLE 2:** Results of multilevel models.

Model	Standardized coefficient (SE)		95% CI	*t*	*df*	*p*
School absence^a^						
Time ME	−0.07	(0.07)	[−0.22; 0.07]	−1.00	279	0.318
Group ME	0.16	(0.15)	[−0.14; 0.46]	1.07	126	0.288
Interaction	0.12	(0.09)	[−0.07; 0.30]	1.25	279	0.212
Days with medication consumption^a^						
Time ME	−0.03	(0.05)	[−0.13; 0.07]	−0.59	279	0.557
Group ME	−0.29	(0.16)	[−0.62; 0.03]	−1.77	126	0.079
Interaction	0.00	(0.07)	[−0.13; 0.13]	−0.04	279	0.970
Days with headaches^a^						
Time ME	0.02	(0.06)	[−0.09; 0.13]	0.43	279	0.667
Group ME	−0.08	(0.16)	[−0.40; 0.24]	−0.51	126	0.608
Interaction	−0.03	(0.07)	[−0.16; 0.11]	−0.37	279	0.712
Maximum headache intensity^a^						
Time ME	−0.09	(0.08)	[−0.25; 0.07]	−1.05	279	0.295
Group ME	0.18	(0.14)	[−0.1; 0.46]	1.26	126	0.210
Interaction	0.03	(0.1)	[−0.17; 0.23]	0.30	279	0.763
Average headache intensity^a^						
Time ME	−0.11	(0.08)	[−0.27; 0.05]	−1.38	279	0.169
Group ME	0.18	(0.15)	[−0.11; 0.47]	1.24	126	0.216
Interaction	0.03	(0.1)	[−0.17; 0.23]	0.31	279	0.760
Recurrent headaches^b^						
Time ME	0.77	(0.12)	[0.57; 1.05]	−1.65	‐	0.099
Group ME	1.21	(0.53)	[0.51; 2.87]	0.43	‐	0.667
Interaction	0.67	(0.14)	[0.45; 1.01]	−1.90	‐	0.057
Headache‐related knowledge						
Time ME	−0.06	(0.02)	[−0.10; −0.01]	−2.57	1296	**0.010**
Group ME	0.50	(0.05)	[0.40; 0.60]	9.44	791	**<0.001**
Interaction	0.35	(0.03)	[0.30; 0.41]	11.78	1296	**<0.001**
Pain self‐efficacy						
Time ME	0.08	(0.02)	[0.04; 0.12]	3.82	1831	**<0.001**
Group ME	0.06	(0.06)	[−0.06; 0.18]	1.04	791	0.300
Interaction	0.03	(0.03)	[−0.03; 0.08]	0.94	1831	0.345
Passive pain coping						
Time ME	0.02	(0.02)	[−0.03; 0.06]	0.75	1835	0.454
Group ME	−0.08	(0.06)	[−0.19; 0.04]	−1.27	791	0.204
Interaction	−0.06	(0.03)	[−0.12; 0.00]	−2.01	1835	**0.044**
Positive self‐instructions						
Time ME	−0.04	(0.02)	[−0.09; 0.00]	−1.91	1835	0.056
Group ME	0.05	(0.06)	[−0.07; 0.17]	0.85	791	0.395
Interaction	−0.03	(0.03)	[−0.09; 0.03]	−0.91	1835	0.362
Seeking social support						
Time ME	−0.04	(0.02)	[−0.08; 0.00]	−1.87	1835	0.062
Group ME	0.04	(0.06)	[−0.08; 0.16]	0.64	791	0.522
Interaction	0	(0.03)	[−0.05; 0.06]	0.11	1835	0.912

*Note*: Observations are nested within students (*N* = 793). Assessments took place before the intervention (T1) and subsequently at 4‐week intervals (T2–T4). Treatment groups were the intervention group (IG) and control group (CG). Reference categories were CG for treatment; T1 was compared to the reference categories T4 (overall treatment effect) and to T2 (intervention effect). *p* < 0.05 are set in bold.

Abbreviations: CI, confidence interval; ME, main effect; SE, standard error.

^a^ Outcome only analyzed for children reporting recurrent headaches at T1 (*n* = 128).

^b^ Dichotomous outcome for which a generalized linear mixed‐effects model was performed; coefficients are odds ratios; test statistics are *z*‐scores.

For all participating children, the IG demonstrated a significantly greater increase in headache‐specific knowledge compared to the CG, indicated by a significant interaction effect of time × group (*β =* 0.35, 95% confidence interval [CI] = [0.30; 0.41], *p* < 0.001). Post hoc tests revealed that the intervention led to a 2.08‐point increase in knowledge score in the IG from T1 to T4 (Supplement S5 in supporting information).

There was a small overall increase in pain self‐efficacy, resulting in a main effect of time (*β* = 0.08, 95% CI = [0.04; 0.12], *p <* 0.001). Post hoc tests revealed that pain self‐efficacy increased significantly from T1 to T4 by 2.1 points (equivalent to 0.19 on the five‐point scale) across both groups. However, no significant time × group interaction was detected.

A significant interaction effect indicated that, over time, students in the IG used fewer passive pain coping strategies compared to those in the CG (time × group interaction: *β =* −0.06, 95% CI = [−0.12; 0.00], *p* = 0.044). From T1 to T4, passive pain coping scores decreased by an additional 0.7 points in the IG compared to the CG (*d* = −0.14; Supplement S5 and Figure 2). For the other two coping subscales, positive self‐instructions and seeking social support, no statistically significant main or interaction effects emerged.

### Subgroup analyses

Including headache recurrence as a third factor in the multilevel models did not reveal statistically significant interaction effects of time × group × headache status across the investigated outcomes (Supplement S6 in supporting information).

In additional analyses restricted to children in the IG (*n* = 414), those who reported revisiting the website at T3 or T4 (*n* = 207) were compared to those who did not (*n* = 207). A total of 81 children in the IG reported recurrent headaches at T1. Of these, 42 revisited the website (51.9%). There were no significant time × revisit interactions (Supplement S7 in supporting information).

### Mediation analysis

**FIGURE 3:**
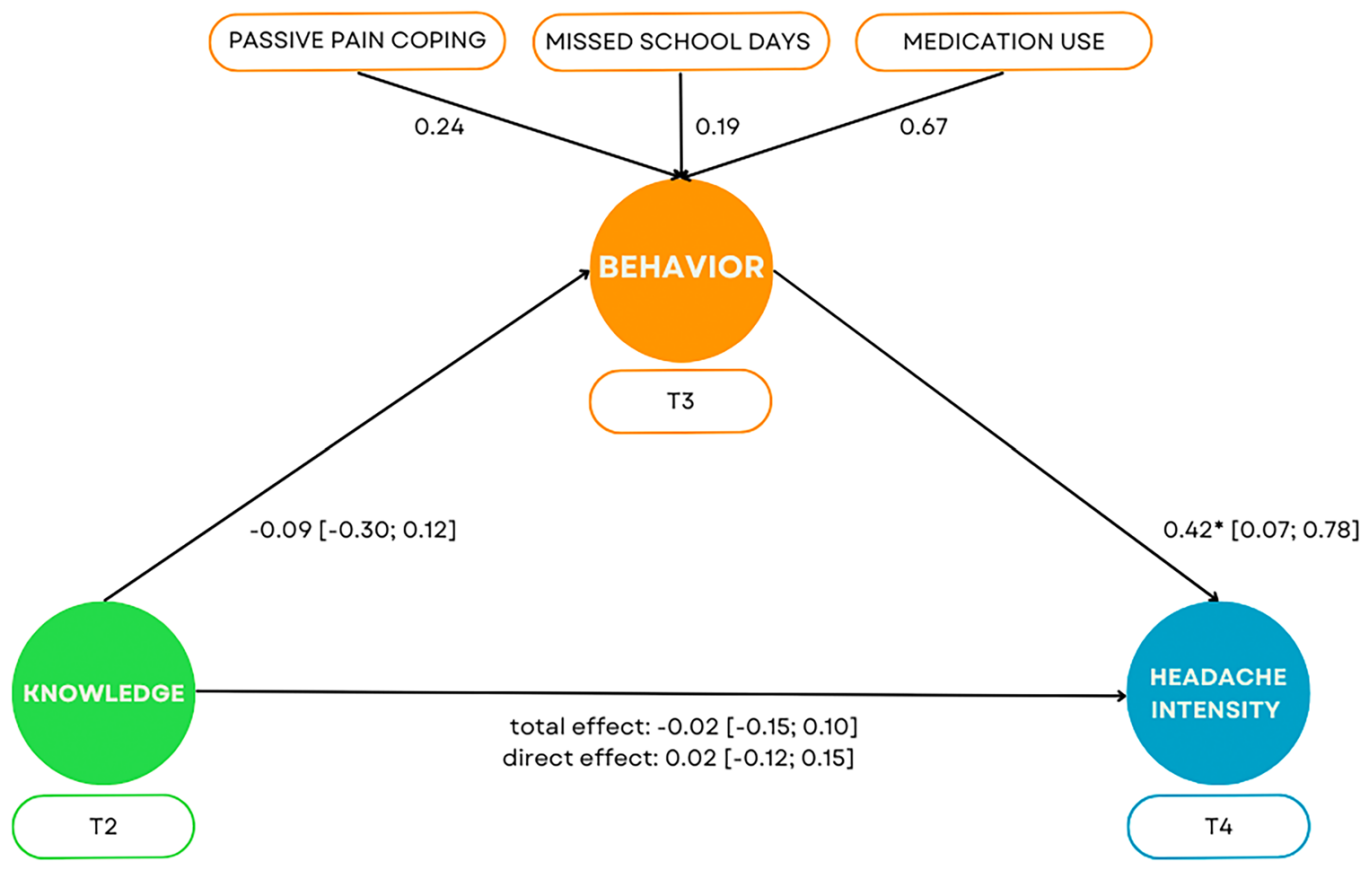
Structural equation model of the relationship among headache‐related knowledge, dysfunctional headache behavior, and average headache intensity. Standardized path coefficients are displayed with their corresponding 95% confidence intervals in square brackets. Standardized factor loadings are presented for pain behavior components. *p < 0.05. T2, T3 and T4; Second, third, and fourth assessment. [Colour figure can be viewed at wileyonlinelibrary.com]

A mediation analysis was conducted to investigate whether headache‐specific knowledge predicted average headache intensity and whether this relationship was mediated by behavior in children with headaches at T1 (Figure 3). There was no direct effect of headache‐specific knowledge on headache intensity (*β* = 0.02, 95% CI [−0.12; 0.15], *p* = 0.796), and this finding did not change when behavioral variables were included as mediators in the model (*β* = −0.02, 95% CI [−0.15; 0.10], *p* = 0.735). Additionally, headache‐specific knowledge did not significantly predict the mediator, *β* = −0.09, 95% CI [−0.30; 0.12], *p* = 0.384. Yet, the behavioral variables did predict headache intensity, *β* = 0.42, 95% CI [0.07; 0.78], *p* = 0.019. Consequently, the relationship between headache‐related knowledge and headache intensity was not mediated by behavior (indirect effect: *β* = −0.04, 95% CI [−0.13; 0.06], *p* = 0.414). Model fit indices indicated good fit: CFI = 1.00, RMSEA [95% CI] = 0.00 [0.00; 0.08], and SRMR = 0.02.

## DISCUSSION

In this RCT, we investigated whether engagement with an educational website and accompanying workbook has a health‐promoting impact on headache knowledge, pain self‐efficacy, behavior, and headache characteristics. The intervention significantly improved headache knowledge, but did not significantly affect headache‐related school absence. Although there was a small reduction in passive pain coping strategies in the IG, no significant differences were detected in other behaviors (school absence, medication use, coping strategies), headache characteristics, or pain self‐efficacy.

The retention of headache‐related knowledge over 3 months demonstrates the intervention's effectiveness as an educational tool, supporting our first hypothesis. These findings align with previous studies indicating that pain‐related education in school settings can improve knowledge.^35^, ^36^ However, as observed in studies across various pain contexts, increasing knowledge alone may not induce behavioral changes.^37^, ^38^ Our third hypothesis was that acquiring headache‐related knowledge would lead to changes in headache‐related behavior, subsequently affecting headache intensity. Yet, our mediation analysis contradicts prior research:^8^ headache‐related knowledge did not predict headache intensity. The inclusion of behavioral variables as mediators did not strengthen this explanatory pathway; notably, only behavior predicted headache intensity. Unlike studies that assessed predictors and outcomes simultaneously,^8^ our study evaluated predictors before outcomes, reducing potential bias and allowing for causal interpretation.^39^ Thus, enhancing knowledge may not necessarily translate into behavioral changes that reduce headache intensity.

Several factors may explain why behavior and headache characteristics were minimally influenced by our educational intervention. The participants' young age (*M* = 10.72 years) may limit their ability to recognize the need for behavioral change to alleviate headaches. A related study found that older students (grades 6–8) demonstrated significant improvements compared to younger students (grades 3–5) due to pain neuroscience education.^40^ Younger children may lack the self‐determination needed to implement behavioral intentions independently; children aged 7 to 10 with chronic pain seek social support more often than adolescents (11–18 years).^41^ Furthermore, involving parents in online interventions may enhance their effectiveness for younger children.^42^

The short follow‐up period in our study (~2.5 months) may also account for the lack of observed changes in outcomes. For instance, one study that conducted a 60‐min headache prevention program in class reported increased headache cessation after 7 months.^43^ Another study on lower back pain observed significantly fewer pain episodes after 9 months.^44^ Longer follow‐ups may be necessary to detect the adoption of new behaviors and lasting effects on headache symptoms.

The reduction in passive pain coping strategies among children in the IG, despite no changes in other behaviors, may stem from the intervention's focus on active headache management. Other behaviors were less emphasized, suggesting that while the core message was conveyed, reinforcement is needed to modify ingrained behaviors.

Our second hypothesis was that children with recurrent headaches before the intervention and those who revisited the website would experience greater benefits. Contrary to our expectations, these factors were not significantly associated with improved intervention effectiveness. This finding contributes to the existing literature that shows inconsistent results: while some studies indicate differential effects,^10^, ^45^ others do not.^8^, ^9^ A more detailed analysis of website visit duration and specific engagement areas could provide insights into the interaction needed to influence outcomes. However, we opted against personalized accounts for each IG student to maintain ease of access and avoid issues with forgotten login information. Future studies should examine how website‐based education affects different subgroups and levels of engagement.

### Strengths and limitations

To our knowledge, this study is the first analysis of the impact of a guided website‐based educational intervention targeting tension‐type headache and migraine in schoolchildren. However, several aspects should be considered when interpreting the results. Intervention intensity likely varied among students; some missed the session and completed the workbook as homework, while others did not finish it during class. Moreover, classroom distractions could have affected engagement with the intervention. Nevertheless, these effects reflect realistic school settings and are likely non‐systematic.

Despite these limitations, classroom interventions offer a valuable opportunity to reach children from diverse social backgrounds, potentially reducing health inequalities. Socioeconomic background influences the quality of health care and literacy. In school, children can be reached regardless of their background. The current study included six schools of varying types, reflecting the German school system and engaging children of different educational levels. The results indicate that the intervention can be integrated effectively into 1.5 h lessons across these educational settings, highlighting its potential for broad implementation.

Fostering knowledge can support health behavior change, but other factors must also be considered. The Integrated Theory of Behavior Change^14^ suggests that knowledge and beliefs influence self‐regulation skills and indirectly affect engagement in self‐management behaviors and health status, but so does social facilitation. Thus, educational interventions may need reinforcement from additional measures to effectively change behaviors and symptoms. In the case of headaches, more intensive medical treatment and greater involvement of the social context might be required for children and adolescents with headaches to achieve significant behavioral and symptomatic improvements.

### Practical implications and outlook

The intervention can serve as an accessible educational tool for schools that is easily integrated into the curriculum. It can help school nurses and psychologists provide timely interventions and potentially prevent inappropriate medication use. The intervention's effectiveness may be enhanced by repeating it in middle and high school settings or incorporating additional features from the Internet Intervention Model,^13^ such as self‐assessments for headache symptoms or more personalized content. Future studies could explore whether the intervention influences pain beliefs and the intention to change behavior, which previous studies have indicated.^36^, ^46^ It is suggested that intentions predict behavior, with action planning moderating this relationship.^47^ Assessing these factors could reveal why students adopt or neglect the health‐promoting knowledge they have acquired. In health‐care settings, the website may be used to improve children's headache knowledge prior to appointments, enabling professionals to focus on individual needs and reinforce learning after the appointment.

## CONCLUSION

The combination of a workbook and website utilized in this study is an effective, cost‐efficient approach to disseminating knowledge about common primary headache types. Future studies should explore how tools like this can more effectively induce behavioral changes in children, translating the knowledge gained into practical applications that alleviate headache symptoms.

## AUTHOR CONTRIBUTIONS

**Henrike Goldstein:** Conceptualization; data curation; formal analysis; investigation; methodology; project administration; validation; visualization; writing – original draft. **Lisa‐Marie Rau:** Data curation; formal analysis; methodology; software; supervision; validation; visualization; writing – review and editing. **Clarissa Humberg:** Data curation; formal analysis; software; writing – review and editing. **Verena Bachhausen:** Investigation; writing – review and editing. **Lorin Stahlschmidt:** Conceptualization; supervision; writing – review and editing. **Julia Wager:** Conceptualization; funding acquisition; investigation; methodology; project administration; resources; supervision; validation; writing – review and editing.

## FUNDING INFORMATION

This study is part of the “Chronic Headache in Adolescents: The Patient Perspective on Health Care Utilization” (CHAP) study supported by the German Federal Ministry of Education and Research (grant number 01GY1615). The funding source had no role in the study's design or execution; collection, management, analysis, and interpretation of data; manuscript preparation; or the decision to submit the manuscript for publication.

## CONFLICT OF INTEREST STATEMENT

**Henrike Goldstein**, **Lisa‐Marie Rau**, **Clarissa Humberg**, **Verena Bachhausen**, **Lorin Stahlschmidt**, and **Julia Wager** declare no conflicts of interest with respect to the research, authorship, or publication of this article.

## CLINICAL TRIALS REGISTRATION NUMBER

The study was preregistered in the German Clinical Trials Register (ID: DRKS00027065).

## Supporting information


File S1.

File S2.

File S3.

File S4.

File S5.

File S6.

File S7.

## Data Availability

Data and program codes are available upon request.
