# Supplementary material for: Efficacy of an educational website on headaches in schoolchildren: A cluster‐randomized controlled trial
Source: Headache. 2025 Mar 14;65(6):961–72. doi: 10.1111/head.14923 (PMC12129248; doi:10.1111/head.14923)
Supplement: Supplementary file 1 — File S1. [file HEAD-65-961-s002.docx]

**Supplementary Material 1**

*Application of the criteria for websites from the Internet Intervention Model^1^* *to the website used in this study*

| **Website features** | **Application to the newly developed website** |
| --- | --- |
| **Appearance** (including the look and feel of the application) | For an appealing look and a high recognition value, we have opted for a bright color scheme and pleasing images and layouts. Recurring companions on the website are the two eggs, one of which stands for migraine and the other for tension-type headache. They appear in various graphics and videos. Good usability on both small and large screens and devices was also important. |
| **Behavioral prescriptions** (including instructions on what to do to address the targeted problem) | Clear instructions for helpful behaviors can be found at various points on the website for various situations. They can be found in texts, captions on images and in videos. They are often in the form of step-by-step instructions.  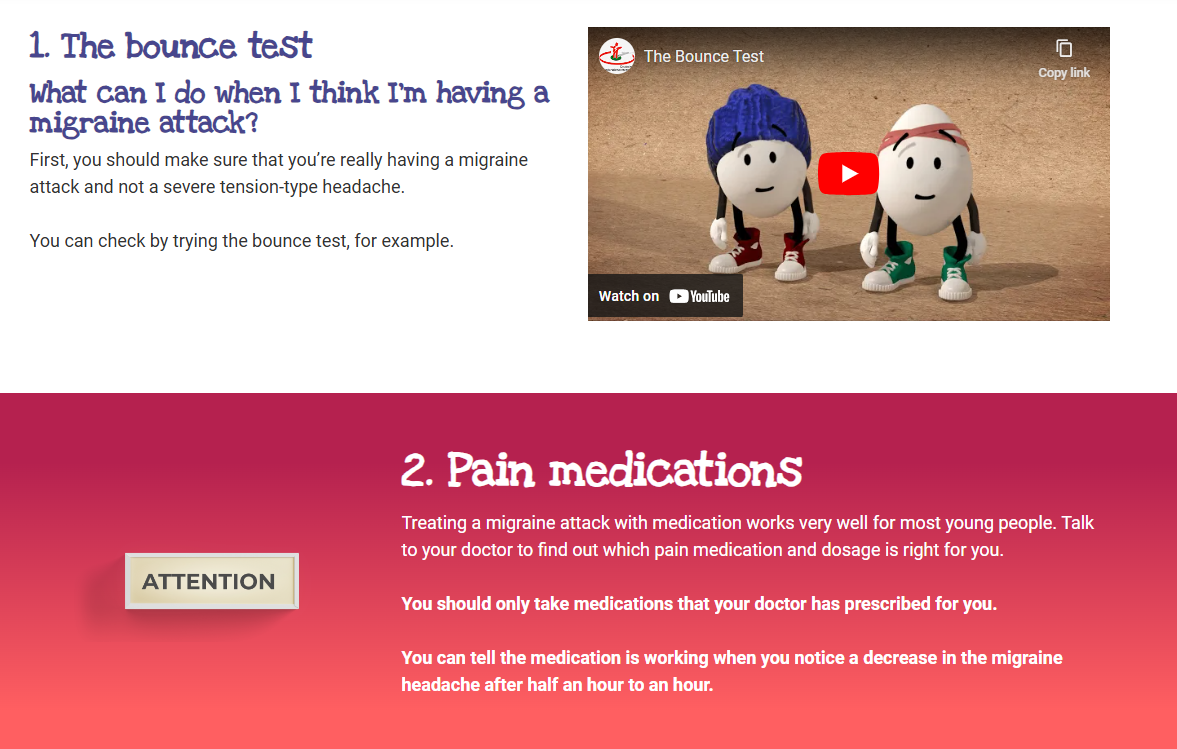 |
| **Burdens** (in terms of website content, length of the intervention and navigation issues) | To keep the burden of the website as low as possible, we kept its structure simple and pretested the website with children of the target population. The thematically related pages are always structured in the same way, there is an intuitive navigation function and a small bar on each page that shows where on the website you are currently located.  We have also kept the information as short and concise as possible. We therefore provide the core information in videos, infographics and short texts. Further information is hidden in drop-down boxes so the pages are not overwhelming and that users may dive deeper if they feel like it.  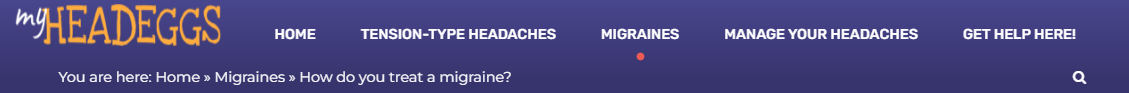 |
| **Content** (including the actual treatment infromation) | The content of the website has been carefully curated by the experts at the German Paediatric Pain Centre. Content and treatment information are evidence-based and guideline-compliant. Accurate, clear and simple language was chosen to best reach the target group and convey the content. |
| **Delivery** (including ways in which the content can be delivered) | The content of the website is conveyed in various ways. The core information can be found in prominently placed videos and in drop-down boxes. Some content is placed on images for clearer illustration. In this case, the texts are also provided as alternative text so that they can also be received by people with visual impairments. There are also interactive elements, e.g. a slider that can be moved to see the effect of taking migraine medication.  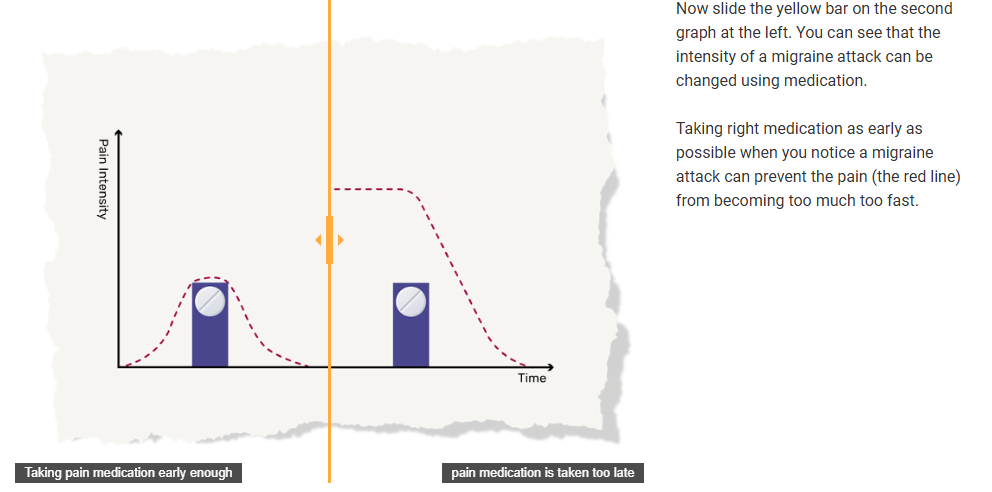 |
| **Message** (including source and style of the content, providing essential information about who created it and how it is presented) | The user can find more information on who created the website on a designated subpage. Here, the people who worked on the project, created the website, translated the content and created the graphics and videos are named. It further informs on how the idea for the website came up and how it was implemented. |
| **Participation** (including engaging and involving the user in the intervention by offering interactive elements, rewards or testings) | Various elements have been incorporated into the website for better user involvement. In terms of interaction, there are, for example, the aforementioned sliders that can be moved on images, or “slot machines“ that provide movement ideas from A to Z at the touch of a button. The sections on tension-type headache and migraine end with quizzes in which one can test their knowledge. Further information is provided after each question.  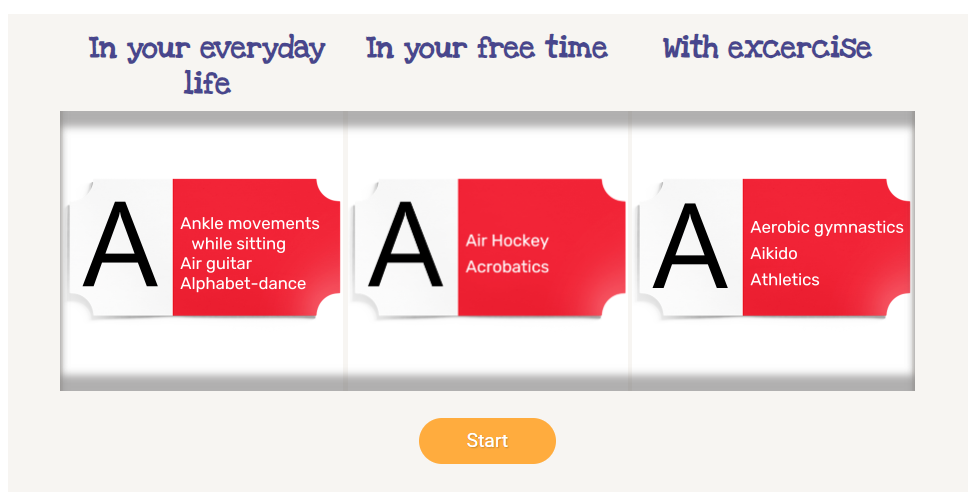 |
| **Assessment** (including measuring the user’s needs, personalization und tailoring of the content) | The sections on a healthy lifestyle (promoting physical activity, stress reduction/ relaxation, and sleep hygiene) each start with a self-check, where you have to tick statements if they apply to you. A picture of an egg that is particularly fit or lazy, for example, then appears. The ticked statements result in a score, which is intended to indicate the extent of the need for improvement in the respective area.  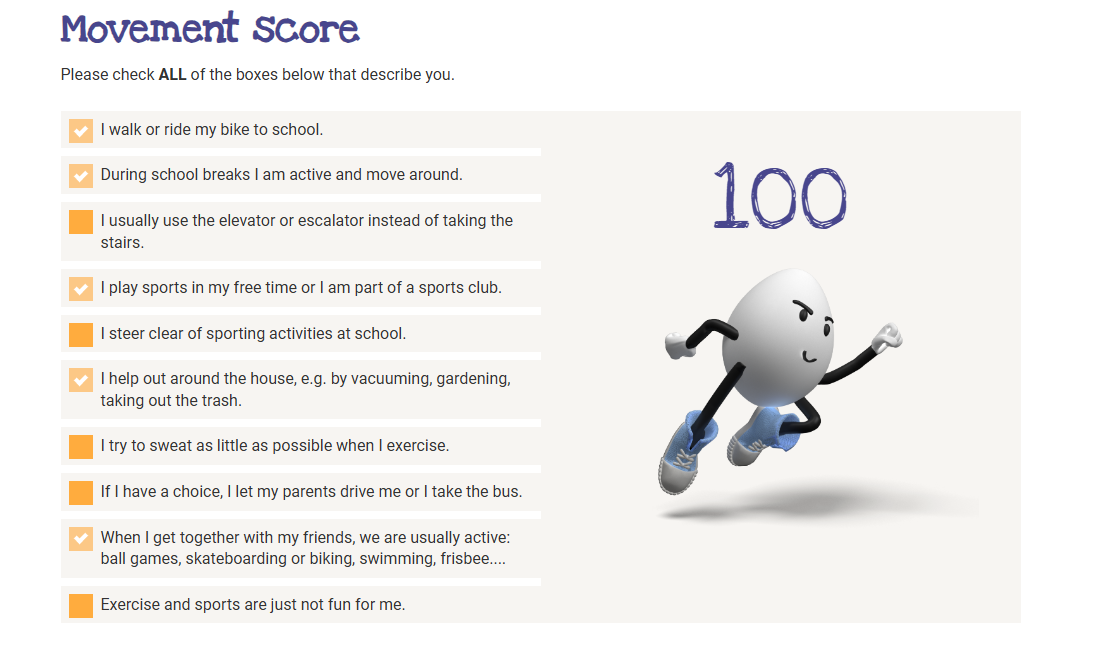 |

*Notes.* The Internet Intervention Model purports that effective Internet interventions produce (and maintain) behavior change and symptom improvement via nine nonlinear steps. One of these steps is the website itself, for which eight factors are described that can be modified for the most robust treatment possible. In this supplement, we describe how these factors were implemented on the website used in this study.

Reference: 1. Ritterband LM, Thorndike FP, Cox DJ, et al. A behavior change model for internet interventions. *Ann Behav Med* 2009; 38: 18–27.
