# Supplementary material for: Efficacy of an educational website on headaches in schoolchildren: A cluster‐randomized controlled trial
Source: Headache. 2025 Mar 14;65(6):961–72. doi: 10.1111/head.14923 (PMC12129248; doi:10.1111/head.14923)
Supplement: Supplementary file 2 — File S2. [file HEAD-65-961-s005.docx]

**Supplementary Material 2**

*Results of the multilevel models including all children with assent (N = 814)*

| **Model** | Standardized coefficient (SE) | | | 95% CI | *t* | *df* | *p* |
| --- | --- | --- | --- | --- | --- | --- | --- |
| **School Absence^a^** | |  |  |  |  |  |  |
| Time ME | | -0.03 | (0.07) | [-0.16;0.11] | -0.37 | 296 | 0.710 |
| Group ME | | 0.17 | (0.14) | [-0.11;0.45] | 1.20 | 135 | 0.232 |
| Interaction | | 0.07 | (0.09) | [-0.11;0.24] | 0.77 | 296 | 0.441 |
| **Days with medication consumption^a^** | |  |  |  |  |  |  |
| Time ME | | -0.04 | (0.05) | [-0.13;0.06] | -0.77 | 296 | 0.444 |
| Group ME | | -0.23 | (0.16) | [-0.54;0.08] | -1.45 | 135 | 0.149 |
| Interaction | | 0.00 | (0.06) | [-0.12;0.13] | 0.04 | 296 | 0.968 |
| **Days with headaches^a^** | |  |  |  |  |  |  |
| Time ME | | 0.01 | (0.05) | [-0.09;0.12] | 0.29 | 296 | 0.771 |
| Group ME | | -0.06 | (0.15) | [-0.37;0.24] | -0.42 | 135 | 0.675 |
| Interaction | | -0.02 | (0.07) | [-0.15;0.11] | -0.25 | 296 | 0.801 |
| **Maximum headache intensity^a^** | |  |  |  |  |  |  |
| Time ME | | -0.08 | (0.07) | [-0.23;0.07] | -1.09 | 296 | 0.275 |
| Group ME | | 0.15 | (0.13) | [-0.12;0.41] | 1.11 | 135 | 0.268 |
| Interaction | | 0.03 | (0.10) | [-0.17;0.22] | 0.27 | 296 | 0.788 |
| **Average headache intensity^a^** | |  |  |  |  |  |  |
| Time ME | | -0.09 | (0.07) | [-0.24;0.05] | -1.29 | 296 | 0.198 |
| Group ME | | 0.15 | (0.14) | [-0.13;0.43] | 1.07 | 135 | 0.284 |
| Interaction | | 0.01 | (0.10) | [-0.17;0.20] | 0.15 | 296 | 0.882 |
| **Headache-related knowledge** |  | |  |  |  |  |  |
| Time ME | -0.05 | | (0.02) | [-0.09;0.00] | -2.16 | 1329 | **0.031** |
| Group ME | 0.51 | | (0.05) | [0.41;0.61] | 9.76 | 812 | **<0.001** |
| Interaction | 0.35 | | (0.03) | [0.29;0.40] | 11.64 | 1329 | **<0.001** |
| **Pain self-efficacy** |  | |  |  |  |  |  |
| Time ME | 0.08 | | (0.02) | [0.04;0.12] | 4.07 | 1875 | **<0.001** |
| Group ME | 0.08 | | (0.06) | [-0.04;0.20] | 1.38 | 812 | 0.169 |
| Interaction | 0.02 | | (0.03) | [-0.03;0.08] | 0.86 | 1875 | 0.391 |
| **Passive Pain Coping** |  | |  |  |  |  |  |
| Time ME | 0.01 | | (0.02) | [-0.03;0.05] | 0.64 | 1879 | 0.525 |
| Group ME | -0.08 | | (0.06) | [-0.20;0.04] | -1.33 | 812 | 0.184 |
| Interaction | -0.06 | | (0.03) | [-0.12;0.00] | -1.95 | 1879 | 0.052 |
| **Positive self-instructions** |  | |  |  |  |  |  |
| Time ME | -0.04 | | (0.02) | [-0.09;0.00] | -1.99 | 1879 | **0.047** |
| Group ME | 0.05 | | (0.06) | [-0.07;0.17] | 0.82 | 812 | 0.410 |
| Interaction | -0.03 | | (0.03) | [-0.09;0.03] | -0.90 | 1879 | 0.367 |
| **Seeking social support** |  | |  |  |  |  |  |
| Time ME | -0.04 | | (0.02) | [-0.08;0.00] | -1.91 | 1879 | 0.057 |
| Group ME | 0.03 | | (0.06) | [-0.08;0.15] | 0.55 | 812 | 0.583 |
| Interaction | 0.00 | | (0.03) | [-0.05;0.06] | 0.12 | 1879 | 0.902 |

*Notes.* The current analyses include children from the control group (CG) who stated that they had visited the website and had thus been excluded from the main analysis. Observations are nested within students (*N* = 814). Assessments took place before the intervention (T1) and subsequently at 4-week intervals (T2 – T4). Treatment groups were the intervention group (IG) and control group (CG). Reference categories were CG for treatment; T1 was compared to the reference categories T4 (overall treatment effect) and to T2 (intervention effect). Outcomes marked with ^a^ were only analyzed for children reporting recurrent headaches at T1 (*n* = 137). *p* < .05 are set in bold. SE = standard error; CI = confidence interval; ME = main effect.
