# Supplementary material for: Efficacy of an educational website on headaches in schoolchildren: A cluster‐randomized controlled trial
Source: Headache. 2025 Mar 14;65(6):961–72. doi: 10.1111/head.14923 (PMC12129248; doi:10.1111/head.14923)
Supplement: Supplementary file 3 — File S3. [file HEAD-65-961-s003.docx]

**Supplementary Material 3**

*Headache-related knowledge questionnaire*

[Questions in italics were excluded from the analysis]

We are interested in what you already know about headaches.

So if you're not sure about any questions, that's no problem. Just answer the questions as best you can. There is only one correct answer per question.

1. *What are the two most common types of headaches?*
   1. *Migraine and exertional headache*
   2. *Tension-type headache and migraine*
   3. *Exertional headache and sleep-related headache*
   4. *Sleep-related headache and tension-type headache*
2. What does a typical migraine attack feel like if you don’t take any medication?
   1. The headache worsens quickly, lasts for a few hours, and then fades away until it stops completely.
   2. The headache varies in intensity, depending on the time of day, and is over within 24 hours.
   3. The headache stays the same and disappears completely after a few weeks.
   4. The headache worsens quickly, lasts for a few minutes, and then goes away.
3. Liam keeps having severe, throbbing headaches. Often, he can’t handle bright light and loud noises. When he walks up stairs, the pain gets worse. He often realizes the day before that a headache is coming.
   Which type of headache does most likely Liam have?
   1. Liam has a tension type headache. They often announce themselves/become noticeable beforehand and intensify with movement or exercise.
   2. Liam has a headache because of a sinus infection. Children have these very often.
   3. Liam has a migraine. They are often accompanied by sensitivity to light and sound and worsen with movement or exercise.
   4. Liam doesn’t have a headache at all. He says he has a headache to be left alone.
4. Alina is having problems at school and her math test is coming up. She has had a mild pressing headache for days. She is constantly arguing with her parents and her sleep has been terrible.
   What signs are there and what could Alina do?
   1. Alina is having a hard time. She should trust that next year will be better.
   2. Alina could be having a migraine attack. She should lie down and rest.
   3. Alina is showing signs of being too stressed. She should try speaking to someone about it and relaxation exercises.
   4. Alina’s signs indicate puberty. She doesn’t need to do anything; in a few years she won’t get headaches anymore.
5. *Lucas’s headaches keep getting worse. He’s tried everything that usually helps with his headaches, but nothing has worked. Even medication isn’t helping. Often, he can’t go to school or meet up with friends because of his headaches.
   What should Lucas do?*
   1. *He should consult a doctor who can examine him and give him advice.*
   2. *He should take more medication.*
   3. *He should continue to rest and drink more water.*
   4. *He should switch schools and go to a school that he enjoys going to.*
6. What usually helps the most with tension-type headache?
   1. Medication and rest
   2. Sleeping a lot and eating lots of fruit
   3. Movement/Exercise and distraction
   4. Drinking lots of water and concentrate on the pain
7. Which three are the most common triggers for a migraine attack?
   1. Boredom, homework, and arguments with friends
   2. Greasy food, long reading, and too many sweets
   3. Changes in the weather, stress, or changes in your daily rhythm
   4. Screen time, car fumes, and running
8. Which answer is correct concerning tension-type headache?
   1. Tension-type headache is caused by too much exercise. You should move as little as possible.
   2. Tension-type headache is very severe and is caused by too much screen time.
   3. Tension-type headache is stabbing/piercing, like bright lightning bolts. It lasts for a few seconds.
   4. Tension-type headache feels like a tight bike helmet and is usually mild to moderate.
9. Mia wants to improve her tennis skills, so she trains almost every day. After training, she usually meets up with friends or goes to her music classes, she is learning to play the guitar and the piano. She also has to study for tests and do her homework. Mia has had a headache for a while and is pretty drained.
   What type of headache do you think Mia has, and what is causing it?
   1. Mia has a migraine because she forgets to drink enough water because of all her commitments.
   2. Mia has a tension-type headache which is often caused by too much stress.
   3. Mia has a tension-type headache caused by the one-sided strain on her right arm from playing tennis and practicing the guitar which has shortened her muscles.
   4. Mia has a migraine because she moves too much.
10. What is the “bounce test” good for when you have a headache?
    1. It helps you realize how strong your headaches are.
    2. If your headache gets worse while jumping, this is a strong indication of a migraine.
    3. If your headache gets worse while jumping, this is a strong indication of a tension-type headache.
    4. You get your brain cells moving again, which helps against headaches.
11. Getting enough sleep can reduce headaches. To get a good night’s sleep, I should…
    1. …stick to regular bedtimes.
    2. …watch a movie while falling asleep.
    3. …eat as late as possible so I don’t wake up because I’m hungry.
    4. …heat the room till its cozy and warm.
12. What does exercising/playing sports have to do with headaches?
    1. Playing sports and exercising helps with pain during a migraine attack.
    2. Playing sports and exercising causes headaches by triggering the production of sadness hormones in the brain.
    3. Playing sports and exercising has nothing to do with headaches.
    4. Playing sports and exercising helps prevent tension-type headache and migraine attacks.
13. Which factors all contribute to the development of tension-type headache?
    1. Feelings, genes, and arguments with friends
    2. Bathing, bad moods, and siblings
    3. Playing sports, allergies, and soda
    4. Chocolate, too little sleep, and weather
14. *What are primary headaches?*
    1. *No other illnesses or injuries are causing the headaches.*
    2. *The headache is particularly severe.*
    3. *It is a headache that occurs for the first time.*
    4. *It is the first time you have seen a doctor for a headache.*
15. Simon is working on his homework and suddenly gets a severe migraine attack. He doesn’t know what to do and asks his mom.
    What advice should Simon’s mom give him?
    1. “Just ignore it and get some exercise.”
    2. “Put your homework aside for now and relax in the sun.”
    3. “Put on some loud music and really work up a sweat dancing.”
    4. “Take some pain medication and rest for a while till the medication works.”
