# Supplementary material for: Efficacy of an educational website on headaches in schoolchildren: A cluster‐randomized controlled trial
Source: Headache. 2025 Mar 14;65(6):961–72. doi: 10.1111/head.14923 (PMC12129248; doi:10.1111/head.14923)
Supplement: Supplementary file 4 — File S4. [file HEAD-65-961-s001.docx]

**Supplementary Material 4**

*Used R Packages*

- compareGroups, version 4.7.0 [9]: Group comparisons
- EMAtools, version 0.1.4 [3]: Effect sizes of post-hoc tests
- haven, version 2.5.2 [12]: Importing and exporting files from SPSS
- janitor, version 2.2.0 [2]: Data cleaning and descriptive statistics
- lavaan, version 0.6-17 [8]: Structural equation models
- lme4, version 1.1-33 [1]: Multilevel models
- nlme, version 3.1-157 [6]: Multilevel models
- parameters, version 0.22.1 [4]: Multilevel models
- patchwork, version 1.1.2 [5]: Graphs
- psych, version 2.3.3 [7]: Graphs
- rstatix, version 0.7.2 [3]: Descriptive statistics
- skimr, version 2.1.5 [10]: Data examination
- tidyverse, version 2.0.0 [11]: Data wrangling, analysis, and graphs

**References**

[1] Bates D, Mächler M, Bolker B, Walker S. Fitting Linear Mixed-Effects Models Using lme4. J. Stat. Soft. 2015;67(1):1–48.

[2] Firke S. janitor: Simple Tools for Examining and Cleaning Dirty Data, 2023. Available at: https://CRAN.R-project.org/package=janitor.

[3] Kleiman E. EMAtools: Data Management Tools for Real-Time Monitoring/Ecological Momentary Assessment Data, 2021. Available at: https://CRAN.R-project.org/package=EMAtools.

[4] Lüdecke D, Ben-Shachar MS, Patil I, Makowski D. Extracting, Computing and Exploring the Parameters of Statistical Models using R. Journal of Open Source Software 2020;5(53):2445.

[5] Pedersen TL. patchwork: The Composer of Plots, 2022. Available at: https://CRAN.R-project.org/package=patchwork.

[6] Pinheiro JC, Bates DM. Mixed-Effects Models in S and S-PLUS. New York: Springer-Verlag, 2000.

[7] Revelle W. psych: Procedures for Psychological, Psychometric, and Personality Research. Evanston, Illinois, 2023. Available at: https://CRAN.R-project.org/package=psych.

[8] Rosseel Y. Lavaan: An R package for structural equation modeling and more. Journal of statistical software 2012;48(2):1–36.

[9] Subirana I, Sanz H, Vila J. Building Bivariate Tables: The compareGroups Package for R. J. Stat. Soft. 2014;57(12):1–16.

[10] Waring E, Quinn M, McNamara A, Eduardo Arino de la Rubia, Zhu H, Ellis S. skimr: Compact and Flexible Summaries of Data, 2022. Available at: https://CRAN.R-project.org/package=skimr.

[11] Wickham H, Averick M, Bryan J, Chang W, McGowan LD, François R, Grolemund G, Hayes A, Henry L, Hester J, Kuhn M, Pedersen TL, Miller E, Bache SM, Müller K, Ooms J, Robinson D, Seidel DP, Spinu V, Takahashi K, Vaughan D, Wilke C, Woo K, Yutani H. Welcome to the tidyverse. Journal of Open Source Software 2019;4(43):1686.

[12] Wickham H, Miller E, Smith D. haven: Import and Export ’SPSS’, ’Stata’ and ’SAS’ Files, 2023. Available at: https://CRAN.R-project.org/package=haven.
