# Supplementary material for: Efficacy of an educational website on headaches in schoolchildren: A cluster‐randomized controlled trial
Source: Headache. 2025 Mar 14;65(6):961–72. doi: 10.1111/head.14923 (PMC12129248; doi:10.1111/head.14923)
Supplement: Supplementary file 5 — File S5. [file HEAD-65-961-s007.docx]

**Supplementary Material 5**

*Post-hoc-tests of multilevel models*

| **Model** | *t* | *df* | *p* | *P adj* | *d* |
| --- | --- | --- | --- | --- | --- |
| **Headache-related knowledge** |  |  |  |  |  |
| Time ME | -2.57 | 1296 | **0.010** |  |  |
| Group ME | 9.44 | 791 | **<0.001** |  |  |
| Interaction | 11.78 | 1296 | **<0.001** |  |  |
| **Post-Hoc-Tests** |  |  |  |  |  |
| T1-T2 | 4.32 | 1294 | **<0.001** | **<0.001** | 0.23 |
| T1-T4 | -2.04 | 1294 | **0.041** | 0.052 | -0.11 |
| Group | -0.96 | 791 | 0.337 | 0.337 | -0.07 |
| T1-T2 x Group | 10.19 | 1294 | **<0.001** | **<0.001** | 0.55 |
| T1-T4 x Group | 12.68 | 1294 | **<0.001** | **<0.001** | 0.69 |
| **Pain self-efficacy** |  |  |  |  |  |
| Time ME | 3.82 | 1831 | **<0.001** |  |  |
| Group ME | 1.04 | 791 | 0.300 |  |  |
| Interaction | 0.94 | 1831 | 0.345 |  |  |
| **Post-Hoc-Tests** |  |  |  |  |  |
| T1-T2 | 1.75 | 1827 | 0.081 | 0.081 | 0.09 |
| T1-T3 | 3.38 | 1827 | **<0.001** | **0.001** | 0.21 |
| T1-T4 | 3.48 | 1827 | **<0.001** | **0.002** | 0.19 |
| **Passive Pain Coping** |  |  |  |  |  |
| Time ME | 0.75 | 1835 | 0.454 |  |  |
| Group ME | -1.27 | 791 | 0.204 |  |  |
| Interaction | -2.01 | 1835 | **0.044** |  |  |
| **Post-Hoc-Tests** |  |  |  |  |  |
| T1-T2 x Group | -1.50 | 1831 | 0.135 | 0.203 | -0.08 |
| T1-T3 x Group | -0.15 | 1831 | 0.879 | 0.879 | -0.02 |
| T1-T4 x Group | -2.48 | 1831 | **0.013** | **0.039** | -0.14 |

*Notes.* Post hoc tests are presented for significant overall tests only. Possible ranges of the variables: knowledge scores 0 to 15, pain self-efficacy scores 0 to 44, passive pain-coping scores 0 to 20. Knowledge was assessed at T1, T2 and T4.

ME, Main Effect
