# Supplementary material for: Efficacy of an educational website on headaches in schoolchildren: A cluster‐randomized controlled trial
Source: Headache. 2025 Mar 14;65(6):961–72. doi: 10.1111/head.14923 (PMC12129248; doi:10.1111/head.14923)
Supplement: Supplementary file 6 — File S6. [file HEAD-65-961-s004.docx]

**Supplementary Material 6**

*Results of the multilevel model time×group×headache status*

| **Model** | Standardized coefficient (SE) | | 95% CI | *t* | *df* | *p* |
| --- | --- | --- | --- | --- | --- | --- |
| **Headache-related knowledge** |  |  |  |  |  |  |
| Time ME | 0.30 | (0.03) | [0.24;0.36] | 10.19 | 877 | **<0.001** |
| Group ME | -0.53 | (0.07) | [-0.67;-0.39] | -7.36 | 537 | **<0.001** |
| Headache Status ME | 0.12 | (0.10) | [-0.07;0.31] | 1.27 | 537 | 0.205 |
| Time×Group | -0.36 | (0.04) | [-0.45;-0.28] | -8.61 | 877 | **<0.001** |
| Group×Headache Status | 0.14 | (0.15) | [-0.16;0.45] | 0.94 | 537 | 0.349 |
| Time×Headache Status | -0.02 | (0.06) | [-0.13;0.09] | -0.29 | 877 | 0.773 |
| Time×Group×Headache Status | 0.03 | (0.09) | [-0.15;0.21] | 0.35 | 877 | 0.727 |
| **Pain self-efficacy** |  |  |  |  |  |  |
| Time ME | 0.09 | (0.03) | [0.04;0.14] | 3.36 | 1246 | **<0.001** |
| Group ME | -0.10 | (0.09) | [-0.27;0.07] | -1.18 | 537 | 0.238 |
| Headache Status ME | -0.23 | (0.11) | [-0.45;-0.01] | -2.01 | 537 | **0.045** |
| Time×Group | 0.02 | (0.04) | [-0.05;0.09] | 0.54 | 1246 | 0.587 |
| Group×Headache Status | 0.03 | (0.18) | [-0.33;0.38] | 0.15 | 537 | 0.879 |
| Time×Headache Status | 0.03 | (0.05) | [-0.06;0.13] | 0.67 | 1246 | 0.502 |
| Time×Group×Headache Status | -0.01 | (0.08) | [-0.16;0.15] | -0.08 | 1246 | 0.940 |
| **Passive Pain Coping** |  |  |  |  |  |  |
| Time ME | -0.03 | (0.03) | [-0.09;0.03] | -1.00 | 1247 | 0.318 |
| Group ME | 0.07 | (0.08) | [-0.10;0.23] | 0.80 | 537 | 0.422 |
| Headache Status ME | 0.11 | (0.11) | [-0.10;0.33] | 1.05 | 537 | 0.294 |
| Time×Group | 0.04 | (0.04) | [-0.04;0.12] | 0.89 | 1247 | 0.374 |
| Group×Headache Status | 0.07 | (0.17) | [-0.27;0.41] | 0.39 | 537 | 0.698 |
| Time×Headache Status | -0.03 | (0.06) | [-0.14;0.08] | -0.50 | 1247 | 0.616 |
| Time×Group×Headache Status | 0.06 | (0.09) | [-0.12;0.23] | 0.65 | 1247 | 0.517 |
| **Positive self-instructions** |  |  |  |  |  |  |
| Time ME | -0.06 | (0.03) | [-0.12;0.00] | -2.03 | 1247 | **0.043** |
| Group ME | -0.06 | (0.08) | [-0.22;0.11] | -0.70 | 537 | 0.483 |
| Headache Status ME | -0.25 | (0.11) | [-0.47;-0.04] | -2.28 | 537 | **0.023** |
| Time×Group | -0.02 | (0.04) | [-0.10;0.06] | -0.46 | 1247 | 0.642 |
| Group×Headache Status | 0.10 | (0.18) | [-0.24;0.45] | 0.59 | 537 | 0.554 |
| Time×Headache Status | -0.03 | (0.06) | [-0.14;0.08] | -0.58 | 1247 | 0.565 |
| Time×Group×Headache Status | 0.13 | (0.09) | [-0.05;0.31] | 1.43 | 1247 | 0.154 |

| **Seeking social support** |  |  |  |  |  |  |
| --- | --- | --- | --- | --- | --- | --- |
| Time ME | -0.04 | (0.03) | [-0.09;0.01] | -1.49 | 1247 | 0.137 |
| Group ME | 0.00 | (0.08) | [-0.16;0.16] | 0.00 | 537 | 0.996 |
| Headache Status ME | -0.22 | (0.11) | [-0.44;-0.01] | -2.03 | 537 | **0.043** |
| Time×Group | 0.00 | (0.04) | [-0.07;0.08] | 0.04 | 1247 | 0.971 |
| Group×Headache Status | -0.05 | (0.18) | [-0.40;0.29] | -0.30 | 537 | 0.763 |
| Time×Headache Status | -0.03 | (0.05) | [-0.13;0.07] | -0.57 | 1247 | 0.571 |
| Time×Group×Headache Status | 0.02 | (0.08) | [-0.14;0.18] | 0.26 | 1247 | 0.797 |

*Notes.* Observations are nested within students (*n* = 541). Assessments took place before the intervention (T1) and subsequently at 4-week intervals (T2 – T4). Treatment groups were the intervention group (IG) and control group (CG). Children without headaches at baseline were compared with those with recurrent headaches. Reference categories were CG for treatment and no headaches at baseline for headache status; T1 was compared to the reference categories T4 (overall treatment effect) and to T2 (intervention effect). *p* < .05 are set in bold. SE = standard error; CI = confidence interval; ME = main effect.
