# Supplementary material for: Efficacy of an educational website on headaches in schoolchildren: A cluster‐randomized controlled trial
Source: Headache. 2025 Mar 14;65(6):961–72. doi: 10.1111/head.14923 (PMC12129248; doi:10.1111/head.14923)
Supplement: Supplementary file 7 — File S7. [file HEAD-65-961-s006.docx]

**Supplementary Material 7**

*Results of the multilevel model comparing children in the intervention group (IG) who did vs did not revisit the website*

| **Model** | Standardized coefficient (SE) | | | 95% CI | *t* | *Df* | *p* |
| --- | --- | --- | --- | --- | --- | --- | --- |
| **School Absence^a^** | |  |  |  |  |  |  |
| Time ME | | 0.04 | (0.06) | [-0.07;0.15] | 0.70 | 177 | 0.483 |
| Visits ME | | -0.09 | (0.09) | [-0.28;0.09] | -1.01 | 79 | 0.314 |
| Interaction | | -0.02 | (0.06) | [-0.13;0.09] | -0.30 | 177 | 0.765 |
| **Days with medication consumption^a^** | |  |  |  |  |  |  |
| Time ME | | -0.05 | (0.04) | [-0.12;0.03] | -1.24 | 177 | 0.218 |
| Visits ME | | -0.06 | (0.09) | [-0.24;0.13] | -0.63 | 79 | 0.531 |
| Interaction | | -0.03 | (0.04) | [-0.11;0.04] | -0.85 | 177 | 0.395 |
| **Days with headaches^a^** | |  |  |  |  |  |  |
| Time ME | | 0.00 | (0.04) | [-0.08;0.07] | -0.10 | 177 | 0.920 |
| Visits ME | | -0.12 | (0.10) | [-0.32;0.07] | -1.29 | 79 | 0.199 |
| Interaction | | -0.03 | (0.04) | [-0.11;0.05] | -0.73 | 177 | 0.468 |
| **Maximum headache intensity^a^** | |  |  |  |  |  |  |
| Time ME | | -0.06 | (0.06) | [-0.17;0.06] | -0.92 | 177 | 0.356 |
| Visits ME | | -0.03 | (0.08) | [-0.20;0.13] | -0.41 | 79 | 0.682 |
| Interaction | | 0.00 | (0.06) | [-0.12;0.12] | -0.01 | 177 | 0.990 |
| **Average headache intensity^a^** | |  |  |  |  |  |  |
| Time ME | | -0.08 | (0.06) | [-0.19;0.03] | -1.37 | 177 | 0.171 |
| Visits ME | | -0.01 | (0.09) | [-0.18;0.17] | -0.10 | 79 | 0.922 |
| Interaction | | 0.00 | (0.06) | [-0.11;0.11] | -0.03 | 177 | 0.979 |
| **Headache-related knowledge** |  | |  |  |  |  |  |
| Time ME | 0.28 | | (0.02) | [0.24;0.32] | 13.06 | 681 | **<0.001** |
| Visits ME | -0.04 | | (0.04) | [-0.11;0.03] | -1.07 | 412 | 0.285 |
| Interaction | -0.03 | | (0.02) | [-0.07;0.01] | -1.33 | 681 | 0.186 |
| **Pain self-efficacy** |  | |  |  |  |  |  |
| Time ME | 0.10 | | (0.02) | [0.06;0.14] | 5.30 | 961 | **<0.001** |
| Visits ME | 0.02 | | (0.04) | [-0.06;0.11] | 0.52 | 412 | 0.605 |
| Interaction | 0.01 | | (0.02) | [-0.03;0.04] | 0.36 | 961 | 0.717 |
| **Passive Pain Coping** |  | |  |  |  |  |  |
| Time ME | -0.04 | | (0.02) | [-0.08;-0.01] | -2.24 | 963 | **0.025** |
| Visits ME | 0.05 | | (0.04) | [-0.04;0.13] | 1.11 | 412 | 0.268 |
| Interaction | -0.01 | | (0.02) | [-0.05;0.03] | -0.46 | 963 | 0.642 |
| **Positive self-instructions** |  | |  |  |  |  |  |
| Time ME | -0.07 | | (0.02) | [-0.11;-0.03] | -3.46 | 963 | **<0.001** |
| Visits ME | 0.16 | | (0.04) | [0.08;0.25] | 3.97 | 412 | **<0.001** |
| Interaction | 0.01 | | (0.02) | [-0.03;0.05] | 0.36 | 963 | 0.720 |
| **Seeking social support** |  | |  |  |  |  |  |
| Time ME | -0.03 | | (0.02) | [-0.07;0.00] | -1.74 | 963 | 0.082 |
| Visits ME | 0.12 | | (0.04) | [0.04;0.20] | 2.96 | 412 | **0.003** |
| Interaction | 0.02 | | (0.02) | [-0.02;0.06] | 0.96 | 963 | 0.335 |

*Notes.* Observations are nested within students (*n* = 414). Assessments took place before the intervention (T1) and subsequently at 4-week intervals (T2 – T4). Reference categories were children from the IG who had not revisited the website; T1 was compared to the reference categories T4 (overall treatment effect) and to T2 (intervention effect). Outcomes marked with ^a^ were only analyzed for children reporting recurrent headaches at T1 (*n* = 81). *p* < .05 are set in bold. SE = standard error; CI = confidence interval; ME = main effect.
